# Positive Aspects of Caregiving Are Associated With Lower Risk of Frailty and Sleep Disruption in the National Study of Caregiving

**DOI:** 10.1093/geroni/igac058

**Published:** 2022-09-02

**Authors:** Alexandra M Wennberg, Loretta R Anderson, Lenis P Chen-Edinboro, Annachiara Cagnin, Lorenzo Pini

**Affiliations:** Unit of Epidemiology, Institutet of Environmental Medicine, Karolinska Institutet, Stockholm, Sweden; Program in Gerontology, Department of Epidemiology and Public Health, University of Maryland, Baltimore, Maryland, USA; School of Health and Applied Human Sciences, University of North Carolina Wilmington, Wilmington, North Carolina, USA; Department of Neuroscience (DNS), University of Padova, Padova, Italy; Padova Neuroscience Center, University of Padova, Padova, Italy

**Keywords:** Nationally representative, Population-based, Positive emotions, Practical burden

## Abstract

**Background and Objectives:**

Older adult caregivers have compounded risk for adverse health outcomes; however, evidence investigating the association between caregiving and frailty has been limited. In the National Study of Caregiving (NSOC), we examined the cross-sectional association between caregiving experiences and frailty and sleep disruption.

**Research Design and Methods:**

We included 621 caregivers aged 65 and older from the 2011 NSOC round. They completed a phone interview, including 36 items about caregiving. Using principal component analysis, we identified 3 caregiving components: general burden, positive emotions, and financial-led burden. Frailty was assessed via low energy, shrinking, weakness, reduced activity, and poor self-rated health. Sleep disruption was assessed with 2 questions regarding sleep interruption and trouble falling back asleep.

**Results:**

In models adjusted for age, sex, education, depression and anxiety symptoms, and medical conditions, positive emotions were associated with a reduced relative risk of frailty (relative risk [RR] = 0.94, 95% confidence interval [CI] 0.90, 0.99) while general burden (proportional odds ratio [POR] = 1.96, 95% CI 1.30, 2.93) and financial-led burden (POR = 1.94, 95% CI 1.22, 3.06) were associated with sleep interruption.

**Discussion and Implications:**

Caregiver burden was associated with increased frailty and sleep interruption. Positive emotions were associated with decreased frailty risk. Interventions aimed at reducing the burden and increasing positive emotions in caregivers may improve frailty outcomes.

**Translational Significance:** It is important to identify modifiable risk factors for frailty and sleep disruption because they are prevalent but modifiable outcomes in older adults. Caregiving is associated with burden and positive aspects. Burden is associated with poorer health outcomes, while positive aspects may protect against them. Among older caregivers, we show that positive emotions associated with caregiving are associated with a lower risk of frailty and sleep disruption, while burden is associated with greater risk. Practitioners should screen for these outcomes, and interventions can be developed to target these aspects of the caregiving experience, potentially decreasing the risk of frailty and sleep disruption.

As the population ages, informal caregiving in families is becoming increasingly common. As of 2019, 53 million U.S. adults reported being a caregiver to someone in the previous 12 months, with over half of caregivers aged 50 or older (National Alliance for Caregiving & AARP, 2020). The caregiving role can be challenging and includes duties, such as assisting with personal care, making care management decisions, and providing emotional support and comfort (Adelman et al., 2014). The physical and mental strain associated with being a caregiver has been termed caregiver burden. However, as a recent review that draws from decades-old theories on caregiver burden posited, the definition of the concept has lacked clarity (Liu et al., 2020). Caregiving components―from burden to rewarding aspects―are multifaceted. Further understanding the central components of caregiving, and recognizing how the different aspects of the experience may relate to health, could provide a better understanding of the risk of health outcomes among caregivers.

Caregiving, particularly burden measures, has been associated with an increased risk of poor physical and mental health outcomes and reduced quality of life (Pinquart & Sørensen, 2007). Caregivers may also have a higher risk of frailty, a chronic condition that primarily affects older adults and is defined as an inability to respond to chronic or acute stressors and maintain homeostasis (Morley et al., 2013; Potier, Degryse, Aubouy et al., 2018). Between 4% and 35% of older caregivers are frail, and between 20% and 60% are prefrail, the prodromal frailty stage (Al Snih et al., 2009; Chang et al., 2015; Máximo et al., 2020; Moreira & Lourenço, 2013; Runzer-Colmenares et al., 2014). However, it is unclear to what extent caregiver experience affects frailty risk, positively or negatively. Smaller case–control (*N* = 79) and cross-sectional (*N* = 148) studies did not find an association between caregiver burden and frailty (Alves et al., 2018; Potier, Degryse, Aubouy et al., 2018). Additionally, a longitudinal study of older spousal caregivers (*N* = 79) found that both frailty status and burden levels remained stable over that time (Potier, Degryse, Bihin et al., 2018).

The association between caregiving and sleep disruption is also critical. More than 60% of spousal caregivers report sleep problems, and they are associated with both caregiver burden and poor health outcomes (Gao et al., 2019), including frailty (Balomenos et al., 2021). Indeed, a recent study in the National Health and Aging Trends (NHATS) cohort showed an association between difficulty initiating sleep and frailty (Liu et al., 2021). Caregivers, particularly those who share a bed or a household with the care recipient, might be disturbed by the care recipient at night and have difficulty sleeping due to the stress of caregiving. In a sample from the National Study of Caregiving (NSOC), 16% of caregivers reported sleep disruption and 10% reported that helping the care recipient disrupted their sleep most nights, and this was associated with a negative emotional burden (Leggett et al., 2018). Additionally, in NSOC, performing more medical-related caregiving tasks was associated with poorer sleep among spousal caregivers caring for persons living with dementia (Polenick et al., 2018). Women spousal caregivers seem to be particularly affected, reporting levels of sleep disruption similar to people diagnosed with insomnia (Wilcox & King, 1999).

Caregiver experience might exist on a spectrum where positive and negative aspects might differentially affect the association with health (Fauziana et al., 2018; Wang et al., 2018). Stress and psychological burden, such as that often experienced by caregivers, has been associated with age-related health outcomes (e.g., frailty; Bachmann et al., 2020). Importantly, it seems that women caregivers are at greater risk of stress- and burden-related negative consequences compared to men (Polenick et al., 2018), and frailty and prefrailty are more common among older women caregivers compared to men (Liu et al., 2021). Chronic stress, though multiple pathways, are associated with premature aging, early death, disability, chronic disease, depression, and poor quality of life (Epel & Lithgow, 2014). This might be particularly salient for older caregivers, because chronic stress in older adults is associated with significantly poorer health, even among noncaregivers (Wright et al., 2015). Therefore, we investigated the cross-sectional association between both positive and negative aspects of caregiving and frailty and sleep outcomes among older caregivers, overall and stratified by sex.

## Method

Data were obtained from the NSOC, a nationally representative study of unpaid and family caregivers. NSOC is a substudy of NHATS, a nationally representative study of Medicare beneficiaries aged 65 and older (Montaquila et al., 2012). NSOC is a periodic study that gathers data on the caregiving experience via a phone interview, and the data in this study were from the 2011 round. NSOC participants were identified by the care recipient (or proxy) as someone providing care (e.g., mobility, self-care, household activities, or medical care management) to a NHATS participant, and up to five caregivers were identified for each participant. If the NHATS participant had more than five caregivers, five were selected at random for participation in NSOC. Our study sample included 621 informal caregivers, representing 5,372,298 caregivers across the United States, aged 65 and older with complete data on age, sex, caregiving experience, frailty, and sleep measures (Supplementary Figure 1). NSOC was approved by the Johns Hopkins Bloomberg School of Public Health Institutional Review Board and is funded by the National Institute on Aging (NIA U01AG032947).

### Independent Variable: Caregiver Experience

In NSOC, caregivers were asked multiple questions about their role as a caregiver and their feelings and experiences with caregiving. These questions pertained to practical care (e.g., helping the care recipient with personal care and transportation), the positive aspects of caregiving (e.g., whether the caregiver enjoys being with the care recipient, whether caregiving makes the caregiver feel more confident in their abilities), and emotional burden of caregiving (e.g., whether caregiving felt like too much or exhausting). We included 36 questions (Table 1) that were consolidated using principal component analysis (PCA) to reduce the dimensionality, and we also used PCA to identify the main axes of the caregiver experience (see the statistical section later for the PCA analysis description). PCA was chosen according to its high capability to infer a low-dimensional space from psychological data (Sperber, 2021). Through this unsupervised approach we aimed to identify the main components of caregiving without imposing preconceived concepts of the aspects of experience or duties. This allowed the data to speak for itself regarding the number of items that were most important, arranged into the number of components that were necessary to adequately describe the data. We aimed to represent both the positive and negative aspects of caregiving without determining beforehand how the items might separate or combine.

**Table 1.** Caregiving Experience Question Items

Items	Survey question
Item 1	Is caregiving emotionally difficult for you?
Item 2	Is caregiving physically difficult for you?
Item 3	Is caregiving financially difficult for you?
Item 4	How emotionally difficult is helping?
Item 5	How physically difficult is helping?
Item 6	How financially difficult is helping?
Item 7	How much has your family disagreed about care?
Item 8	Do your friends and family help you with the care?
Item 9	Are you exhausted when you go to bed at night?
Item 10	Do you feel you have more to do than you can handle?
Item 11	Do you feel you don’t have time for yourself?
Item 12	Do you feel as soon as you get a routine going, the care recipient’s needs change?
Item 13	How much does the care recipient argue with you?
Item 14	How much does the care recipient get on your nerves?
Item 15	How often do you help the care recipient with chores around the house?
Item 16	How often do you help the care recipient do their shopping?
Item 17	How often do you help the care recipient order their medications?
Item 18	How often do you help the care recipient pay or manage their bills?
Item 19	How often do you help the care recipient with personal care?
Item 20	Have you helped the care recipient get mobility devices?
Item 21	Have you driven the care recipient around?
Item 22	Have you helped the care recipient coordinate other transport?
Item 23	Have you helped the care recipient keep track of medications?
Item 24	Have you helped the care recipient with exercises?
Item 25	Do you help the care recipient with a special diet or nutrition?
Item 26	Do you help the care recipient get around their home?
Item 27	Have you helped the care recipient manage medical tasks?
Item 28	Have you helped the care recipient speak with their doctor?
Item 29	Have you helped the care recipient coordinate medical care?
Item 30	Have you helped the care recipient with their insurance?
Item 31	Have you helped the care recipient with other health insurance?
Item 32	Does caregiving help you feel appreciated?
Item 33	Does caregiving help you feel confident in your abilities?
Item 34	Has helping the care recipient taught you to deal better with difficult situations?
Item 35	Has helping the care recipient helped you feel closer to them?
Item 36	Has helping the care recipient helped you feel more satisfied?

### Dependent Variables: Frailty Measure and Sleep Quality

The assessment of frailty was based on the frailty measure used in NHATS (Bandeen-Roche et al., 2015) and adapted for the NSOC phone survey data, similar to clinical self-report frailty scores (Ma, 2019). The frailty measure consisted of five items, including low energy, shrinking, weakness, reduced activity, and poor self-rated health, all of which were coded as binary (“0” vs “1”) variables. Low energy was assessed with the question, “In the last month, did you have low energy or were you easily exhausted?” and responses were “yes (1)” or “no (0).” Shrinking was assessed with (a) self-reported height and weight, which were used to calculate body mass index (BMI; kg/m^2^), (b) the questions, “Have you lost 10 or more pounds in the last 12 months?” and (c) “Were you trying to lose weight?” If caregivers had a BMI of less than 18.5 or answered “yes” and “no,” respectively, to questions (b) and (c) regarding weight, then they received a “1” for the shrinking item. Weakness was assessed using four questions pertaining to weakness in the upper and lower body. First, caregivers were asked, “In the last month, did you have limited strength or movement in your (shoulders, arms, or hands)/(hips, legs, knees, or feet)?” If they responded “yes,” they were additionally asked how often it limited their activity (“every day,” “most days,” “some days,” “rarely,” and “never”). If caregivers reported upper or lower body weakness affecting their activity some, most, or every day, they were coded as “1” on weakness, and otherwise were coded as “0.” Lack of activity was based on a “yes (0)” or “no (1)” response question that inquired about caregivers’ participation in physical or group activities (e.g., dinner or bridge clubs, neighborhood or political organizations, and knitting or regular exercise groups) in the last month. Self-rated health asked caregivers to rate their health as “excellent,” “very good,” “good,” “fair,” or “poor”; caregivers with fair/poor health were scored as “1” and otherwise as “0.” Frailty as an outcome was considered as the sum of the items (0–5), where a higher sum represented a greater level of frailty.

Sleep disruption was assessed with two different questions: “In the last month, on nights when you woke up before you wanted to, how often did you have trouble falling back asleep?” and “In the last month, how often did helping the care recipient cause your sleep to be interrupted?” Responses included, “every night,” “most nights,” “some nights,” “rarely,” and “never” (coded from 0 to 4, with 4 being worse). The question about sleep interruption applied only to caregivers who slept in the same household as the care recipient (*n* = 419).

### Covariates

Caregivers provided information on sex, age, and education (coded as <high school, high school degree, and >high school). Depressive symptoms were assessed with short forms of the Patient Health Questionnaire (PHQ-2); scores ranged from 0 to 6 with higher scores indicating greater depressive symptomatology (Kroenke et al., 2003). Anxiety symptoms were measured with the Generalized Anxiety Disorder Scale (GAD-2); scores ranged from 0 to 6 with higher scores indicating greater anxiety (Wild et al., 2013). Caregivers were additionally asked whether a physician had ever told them they had certain medical conditions (i.e., heart attack, heart disease, hypertension, arthritis, osteoporosis, diabetes, lung disease, cancer, and vision and hearing impairment). Presence or absence of each disease was coded as a binary variable (yes or no), and a medical comorbidities count variable was created by summing these conditions.

### Statistical Analysis

All analyses were conducted using round-specific survey weights to create nationally representative estimates. This weighting procedure also accounts for the differential probabilities of selection and nonresponse. We further accounted for clustering and geographic stratification using NSOC variables (Freedman et al., 2020).

To create the caregiver experience scores, we included 36 questions regarding the caregiver’s duties and experience caring for the care recipient in a PCA (Table 1). We expected the components to be correlated, so an oblique rotation was used. Components had to satisfy two criteria: (a) the eigenvalues had to be >1; and (b) the percentage of variance accounted for had to be >5% (Supplementary Table 1).

We then used survey-weighted multivariable-adjusted Poisson regression models to investigate the association between caregiver experience scores and the frailty measure. We used multivariable-adjusted ordinal logistic regression models to investigate the association between (a) caregiver experience scores and trouble falling back asleep; and, separately, (b) caregiver experience scores and interrupted sleep. The proportional odds assumption was checked postestimation, and it was found it was not violated. Model 1 was adjusted for age and sex. Model 2 was adjusted for age, sex, education level, medical conditions, and depressive and anxiety symptoms. We subsequently stratified the analyses by sex because past work has shown that the sex of the caregiver greatly affects caregiver experience, as well as frailty and sleep outcomes (Balomenos et al., 2021; Santos-Orlandi et al., 2017). All analyses were conducted using Stata version 16.0 (StataCorp, College Station, TX).

## Results

The weighted sample had proportionally more women (55.3%) than men (44.6%; Table 2). Caregivers had an overall mean age of 73.7 years (standard error [*SE*]  = 0.32), and men (mean = 74.6 years, *SE* = 0.48) were slightly older than women (mean = 72.9 years, *SE* = 0.43). Women (mean = 2.13, *SE* = 0.09) reported greater depressive symptomatology than men (mean = 1.83, *SE* = 0.08). However, women reported fewer comorbidities (mean = 2.28, *SE* = 0.10) than men (mean = 2.47, *SE* = 0.13).

**Table 2.** Characteristics of Caregivers Aged 65 and Older From Weighted Sample, *n* = 621

Variable	All	Men (44.6)	Women (55.3)
Age, mean (*SE*)	73.7 (0.32)	74.6 (0.48)	72.9 (0.43)
PHQ-2, mean (*SE*)	1.98 (0.06)	1.98 (0.09)	1.99 (0.08)
GAD-2, mean (*SE*)	2.00 (0.06)	1.83 (0.08)	2.13 (0.09)
Comorbidities sum, mean (*SE*)	2.36 (0.08)	2.47 (0.13)	2.28 (0.10)
Frailty score sum, mean (*SE*)	1.43 (0.06)	1.29 (0.09)	1.54 (0.08)
Exhaustion, proportion	29.7	25.2	33.3
Shrinking, proportion	15.7	14.7	16.5
Weakness, proportion	32.7	31.5	33.5
Activity, proportion	40.8	33.8	46.2
Poor health, proportion	23.4	22.7	23.9
Sleep trouble, mean (*SE*)	2.38 (0.05)	2.39 (0.09)	2.38 (0.07)
Sleep interruption,^a^ mean (*SE*)	1.78 (0.06)	1.63 (0.08)	1.89 (0.09)

*Notes: SE* = standard error; PHQ-2 = 2 item Patient Health Questionnaire for depressive symptoms; GAD-2 = 2 item Generalized Anxiety Disorder questionnaire.

^a^Sleep interruption available in a subset of 419 participants (men, *n* = 173; women, *n* = 246).

Of the frailty measure items, not participating in activities was the most common (40.8%), being far more common in women (46.2%) than men (33.8%; Table 2). A greater proportion of women than men endorsed every item of the five included on the frailty measure, indicating greater frailty. Responses to the question about trouble falling back asleep were similar between women (mean = 2.38, *SE* = 0.07) and men (mean = 2.39, *SE* = 0.09); however, among participants who responded to the question about sleep interruption due to helping the care recipient, women reported more disruption (mean = 1.89, *SE* = 0.09) compared to men (mean = 1.63, *SE* = 0.08).

**Figure 1.**
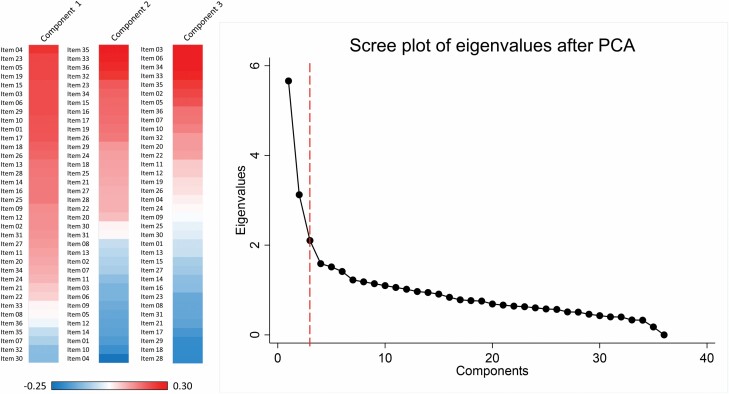
Heat plot of variable loadings for each component with a scree plot of eigenvalues after principal component analysis (PCA) of caregiving experience variables.

From the PCA, we included three caregiving components explaining more than 30% of the total variance (Figure 1). Based on the factor loadings and pattern of each component, we named the first component “general burden,” which was characterized by a broad number of items covering aspects of physical, emotional, and economic difficulty of providing care. This component mainly included 11 items regarding helping the care recipient with personal care, medical care, and household tasks; the component also included feeling that the act of caregiving was physically, emotionally, and practically difficult. The second component was mainly defined by positive experiences with care (e.g., enjoyment being with the care recipient, feelings of closeness to them, and feeling more confident in one’s abilities). Thus, this component was named “positive emotions.” The third component was called “financial-led burden,” because questions relating to financial burden and financial difficulty loaded most heavily onto it, with additional contributions from questions about physical burden.

In Poisson regression models adjusted for age and sex (model 1), general burden (relative risk [RR] = 1.09, 95% confidence interval [CI] 1.05, 1.12) and financial-led burden (RR = 1.08, 95% CI 1.02, 1.15) were associated with a greater relative risk of frailty (Table 3). These associations attenuated toward the null after adjusting for education, depressive and anxiety symptoms, and medical conditions (model 2). In comparison, positive emotions were associated with the reduced relative risk of frailty in both models 1 (RR = 0.93, 95% CI 0.90, 0.98) and 2 (RR = 0.94, 95% CI 0.90, 0.99).

**Table 3.** Association Between Caregiver Experience Components and Frailty and Sleep Outcomes From Poisson (Frailty) and Ordinal Logistic^a^ (Sleep) Regression Models

Outcome measures	Model 1		Model 2	
	RR (95% CI)	POR (95% CI)	RR (95% CI)	POR (95% CI)
Frailty measure outcome				
General burden	1.09 (1.05, 1.12)		1.02 (0.97, 1.08)	
Positive emotions	0.93 (0.90, 0.98)		0.94 (0.90, 0.99)	
Financial-led burden	1.08 (1.02, 1.15)		1.06 (0.96, 1.17)	
Sleep interruption outcome				
General burden		1.63 (1.44, 1.85)		1.96 (1.30, 2.93)
Positive emotions		1.03 (0.89, 1.18)		0.81 (0.61, 1.07)
Financial-led burden		1.25 (1.03, 1.52)		1.94 (1.22, 3.06)
Trouble falling back asleep				
General burden		1.08 (1.00, 1.17)		1.10 (0.94, 1.28)
Positive emotions		0.96 (0.87, 1.05)		1.02 (0.86, 1.21)
Financial-led burden		1.14 (0.98, 1.31)		1.09 (0.85, 1.40)

*Notes:* RR = relative risk; CI = confidence interval; POR = proportional odds ratio. Model 1 adjusted for age and sex, *n* = 621; population, *n* = 5,372,298. Model 2 adjusted for age, sex, education, depression and anxiety symptoms, and medical conditions, *n* = 278; population, *n* = 2,362,341.

^a^Proportional odds assumption not violated.

In the models examining subjective sleep quality outcomes, general burden (model 1: proportional odds ratio [POR] = 1.63, 95% CI 1.44, 1.85; model 2: POR = 1.96, 95% CI 1.30, 2.93) and financial-led burden (model 1: POR = 1.25, 95% CI 1.03, 1.52; model 2: POR = 1.94, 95% CI 1.22, 3.06) were associated with sleep interruption. Similarly, the general burden (POR = 1.08, 95% CI 1.00, 1.17) was associated with trouble falling back asleep, but this association attenuated toward the null after adjusting for additional covariates (model 2).

In age-adjusted sex-stratified models, the association between general burden and frailty was slightly stronger among men (RR = 1.12, 95% CI 1.06, 1.19) compared to women (RR = 1.07, 95% CI 1.03, 1.11; Table 4). These associations attenuated toward the null after adjusting for additional covariates. Among women in model 1, financial-led burden (RR = 1.07, 95% CI 1.00, 1.15) and positive emotions (RR = 0.93, 85% CI 0.89, 0.97) were associated with frailty. In model 2, only the association between positive emotions and frailty remained (RR = 0.94, 95% CI 0.89, 0.99; Table 4). This association was not observed among men.

**Table 4.** Association Between Caregiver Experience Components and Frailty and Sleep Outcomes From Poisson (Frailty) and Ordinal Logistic^a^ (Sleep) Regression Models, by Sex

Outcome measures	Model 1				Model 2			
	Men		Women		Men			Women
	RR (95% CI)	POR (95% CI)	RR (95% CI)	POR (95% CI)	RR (95% CI)	POR (95% CI)	RR (95% CI)	POR (95% CI)
Frailty measure outcome								
General burden	1.12 (1.06, 1.19)		1.07 (1.03, 1.11)		1.07 (0.99, 1.15)		0.99 (0.94, 1.06)	
Positive emotions	0.96 (0.88, 1.04)		0.93 (0.89, 0.97)		0.94 (0.85, 1.03)		0.94 (0.89, 0.99)	
Financial-led burden	1.10 (0.99, 1.22)		1.07 (1.00, 1.15)		0.97 (0.86, 1.10)		1.09 (0.96, 1.24)	
Sleep interruption								
General burden		1.71 (1.43, 2.06)		1.57 (1.31, 1.88)		1.81 (0.91, 3.59)		2.47 (1.44, 4.24)
Positive emotions		0.91 (0.72, 1.13)		1.08 (0.91, 1.28)		1.44 (0.67, 3.11)		0.78 (0.61, 1.00)
Financial-led burden		1.16 (0.86, 1.55)		1.35 (1.07, 1.70)		19.62 (2.04, 188.94)		1.88 (1.17, 3.02)
Trouble falling back asleep								
General burden		0.88 (0.77, 1.00)		1.25 (1.14, 1.37)		0.97 (0.75, 1.25)		1.18 (1.00, 1.39)
Positive emotions		0.95 (0.79, 1.16)		0.96 (0.87, 1.06)		0.93 (0.66, 1.30)		1.09 (0.92, 1.30)
Financial-led burden		1.28 (1.04, 1.59)		1.03 (0.86, 1.03)		1.40 (0.89, 2.20)		0.94 (0.69, 1.27)

*Notes:* RR = relative risk; CI = confidence interval; POR = proportional odds ratio. Model 1 adjusted for age. Frailty: men, *n* = 245; population, *n* = 6,232,601; women, *n* = 376; population, *n* = 3,029,319. Sleep interruption: men, *n* = 173; population, *n* = 1,730,225; women, *n* = 248; population, *n* = 1,671,367. Sleep trouble: men, *n* = 245; population, *n* = 2,342,980; women, *n* = 378; population, *n* = 3,037,466.

Model 2 adjusted for age, education, depression and anxiety symptoms, and medical conditions. Frailty: men, *n* = 92; population, *n* = 759,709; women, *n* = 186; population, *n* = 1,602,631. Sleep interruption: men, *n* = 25; population, *n* = 189,360; women, *n* = 64; population, *n* = 296,251. Sleep trouble: men, *n* = 92; population, *n* = 759,709; women, *n* = 186; population, *n* = 1,602,631.

^a^Proportional odds assumption not violated.

Among men, the general burden was associated with sleep interruption in model 1 (POR = 1.71, 95% CI 1.43, 2.06), while the financial-led burden was associated with sleep interruption in model 2 (RR = 19.62, 95% CI 2.04, 188.94). Among women, general (model 1: POR = 1.57, 95% CI 1.31, 1.88; model 2: POR = 2.47, 95% CI 1.44, 4.24) and financial-led (model 1: POR = 1.35, 95% CI 1.07, 1.70; model 2: POR = 1.88, 95% CI 1.17, 3.02) burden were associated with sleep interruption.

Furthermore, in model 1, the general burden was more strongly associated with trouble falling back asleep in women (POR = 1.25, 95% CI 1.14, 1.37) than men (POR = 0.88, 95% CI 0.77, 1.00), while financial-led burden was associated with trouble falling back asleep in men (POR = 1.28, 95% CI 1.04, 1.59) but not women (POR = 1.03, 95% CI 0.86, 1.03). After adjusting for additional covariates, a general burden in women (POR = 1.18, 95% CI 1.00, 1.39) remained associated with trouble falling back asleep.

In sensitivity analyses, we further adjusted for the relationship between the caregiver and care recipient and whether the caregiver and care recipient lived together, but we did not find that the addition of either of these covariates affected the outcomes (results not shown). We did not adjust for other care recipient variables, as these were considered mediators or independent risk factors for the exposure variable.

## Discussion

In this cross-sectional study, we investigated different aspects of the caregiver experience in a large sample of informal caregivers aged 65 and older. We showed that overall burden and aspects of physical and financial caregiving burden were associated with frailty and sleep disruption, but that these associations trended toward the null after adjusting for depressive and anxiety symptoms and medical conditions. By contrast, the positive emotions associated with caregiving were associated with reduced odds of frailty and sleep disruption, even after adjusting for covariates. In sex-stratified analyses, women showed a stronger association between positive emotions and lower odds of frailty and between measures of burden and poorer sleep outcomes.

In comparison to past studies examining caregiver experience and frailty, this study aimed to examine different aspects of the caregiving experience. We included items about the range of caregiving―both positive and negative, relating to physical, emotional, financial, and relational aspects of caregiving. We found that general burden (characterized by items corresponding to the physical, emotional, and economic difficulty of providing care) and financial-led burden (most strongly characterized by items relating to financial difficulty) were associated with frailty and trouble falling back asleep only prior to adjusting for depression and anxiety symptoms and medical conditions. These results echo findings from smaller case–control and cross-sectional studies (Alves et al., 2018; Potier, Degryse, Aubouy et al., 2018). The finding that depressive symptoms and medical conditions were common in our sample is consistent with findings in caregiving literature (Pinquart & Sørensen, 2007). Together, these findings suggest that the mental health (e.g., depression, anxiety, and loneliness) and physical comorbidities that older caregivers manage might be more predictive of frailty and sleep disruption than the duties of caregiving.

In addition to burden, this study pointed out the positive aspects of caregiving, which were associated with lower frailty score and sleep disruption, particularly among women. Positive aspects of caregiving included being appreciated by care recipients and feeling closer to them. These aspects of caregiving also included feelings of enhanced self-efficacy because of caregiving, including increased confidence in one’s abilities, greater ability to deal with difficult situations, and overall greater satisfaction from caregiving. These potential benefits may also counteract potential feelings of shame or overwhelm associated with a financial burden, which is also associated with stress connected to a scarcity mindset (Adelman et al., 2014). Financial burden (implied in the financial-led component here) has been linked with a number of mental health outcomes and sleep disturbance, which seems to be even more critical during the recent pandemic, and indicates a greater need to address these issues (Beach et al., 2021). This suggests that combating these negative attitudes of caregiving and promoting positive emotions and confidence in one’s caregiving abilities should be cultivated in interventions and support programs for caregivers. Indeed, studies have shown that fostering acceptance of adverse experiences, and defining and committing to personal values, can promote positive appraisal and better coping strategies (Cheng et al., 2019).

Another study in NSOC showed a similar concept, that caregivers who reported a better relationship with the care recipient had better self-reported health (Moon et al., 2017). Studies have shown that framing (i.e., shifting one’s mindset) can affect outcomes and that problem-focused coping, like seeking alternative solutions, choosing, and acting, are associated with better outcomes. Guiding caregivers to address the challenges and responsibilities of caregiving from strengths-based and empowerment-oriented frameworks allows them to accentuate individual strengths, environmental resources, and support strategies that leverage education, social networks, and structural resources (Ponnuswami et al., 2012). Moreover, these approaches foster resiliency, providing caregivers, and by extension care recipients, with an improved sense of competency, coping strategies, ability to plan, and endurance for what could be months or years of the caregiving dynamic (Scholz et al., 2012).

This study had several strengths, including a relatively large, nationally representative sample of caregivers with data on many aspects of caregiving and health. However, the findings must also be considered within the study’s limitations. First, this study is cross-sectional, thus limiting the ability to infer causality. We hypothesized that caregiver experience is associated with frailty measures and sleep, but it is likely that this relationship is bidirectional or cyclical. Future longitudinal studies will be necessary to determine the strength and direction of these associations. From 2021, NSOC data will be collected annually, allowing for more comprehensive data better suited for longitudinal analysis. Second, both sleep questions were subjective sleep assessments, and sleep interruption was available only in a subset of caregivers, limiting power. Although subjective sleep quality is associated with poor health outcomes (Gao et al., 2019), future studies should consider using objective sleep measures. We were also unable to adjust for sleep medication use, which may have affected subjective sleep quality. Similarly, the frailty measure approximated the Fried frailty index used in other NHATS papers (Bandeen-Roche et al., 2015) and was adapted to NSOC because the interviews were conducted over the phone. Therefore, the associations may be slightly more robust if objective measures of strength and weakness were used, as they tend to be more accurate and sensitive.

Here we used PCA to describe the caregiver experience in a low-dimensional space. Although many items were used to help identify different aspects of the caregiving experience, caregiving is multifaceted and is likely not fully captured by 36 items, although this method attempted to represent a fuller spectrum. We drew from a recent review (Liu et al., 2020) that draws upon the foundational theories of caregiving research and, we believe, provides a more clarified concept of the caregiving experience. This approach allowed us to define three main components, explaining more than 30% of the variance of the frailty measure. The first component captured a “general pattern,” consistent with the assumption that the first component best approximates the data in the least squares sense. The second component, relative to the first and third, described the “positive” caregiver experience. The third component represents a less clear pattern with the highest loadings represented by financial and, secondarily, physical burden items. This combination of items in a component is consistent with a recent publication reporting an association between financial burden and physical activity among older adults (Komazawa et al., 2021) and may be directly related to the activity participation component in our frailty measure. The study by Komazawa et al. (2021) showed physical activity, as it relates to physical function, is affected by financial burden. Here the link between financial burden and physical burden may be affected by physical function; or those with financial burden who also receive less instrumental and social support may have reduced physical function. However, we appreciate that our results should be interpreted with caution, and they may not be directly generalizable to other studies of caregiving experience in NSOC or other data sets. Other studies are needed to validate these findings; however, this result might introduce new concepts for interventions aimed at reducing frailty risk in caregivers, which affects both caregiver and care recipient as it may affect the caregiver’s ability to continue in their role.

Overall, we found that measures of negative aspects of the caregiving experience (e.g., physical burden) are positively associated with frailty and sleep disturbance in caregivers, while positive emotions are negatively associated with these outcomes. There is some suggestion for differences between women and men in the strength of these associations. This work may suggest that support and interventions for caregivers should not only aim to reduce the burden but simultaneously enhance the positive emotions associated with caregiving, which could improve physical function outcomes. These approaches have been shown to improve outcomes such as sleep and depression symptoms in caregivers (McCurry et al., 2017; Teri et al., 2005), but may also extend to reducing the risk of frailty. These types of interventions may benefit both caregiver and care recipient, because a healthier caregiver will provide better care management. However, additional research is needed to examine longitudinal trends.

## Supplementary Material

igac058_suppl_Supplementary_Material
