# Supplementary material for: Positive Aspects of Caregiving Are Associated With Lower Risk of Frailty and Sleep Disruption in the National Study of Caregiving
Source: Innov Aging. 2022 Sep 2;6(7):igac058. doi: 10.1093/geroni/igac058 (PMC9579720; doi:10.1093/geroni/igac058)
Supplement: igac058_suppl_Supplementary_Material [file igac058_suppl_supplementary_material.docx]

Online Supplementary Material

Supplementary Figure 1. Flow chart of included participants


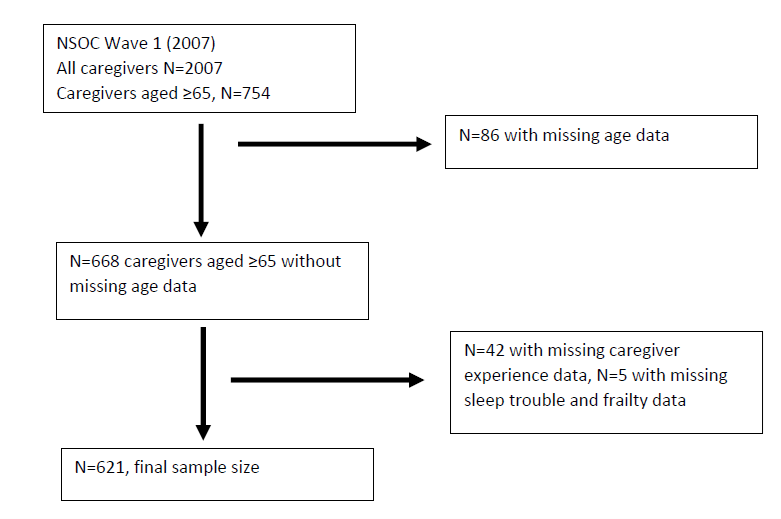


| Supplementary Table 1. Principal component eigenvalues for each component | | | | |
| --- | --- | --- | --- | --- |
| Component | Eigenvalue | Difference | Proportion | Cumulative |
| **Comp1** | **4.81167** | **1.87838** | **0.1504** | **0.1504** |
| **Comp2** | **2.93329** | **0.630452** | **0.0917** | **0.242** |
| **Comp3** | **2.30284** | **0.630633** | **0.072** | **0.314** |
| Comp4 | 1.6722 | 0.104898 | 0.0523 | 0.3662 |
| Comp5 | 1.56731 | 0.144246 | 0.049 | 0.4152 |
| Comp6 | 1.42306 | 0.125822 | 0.0445 | 0.4597 |
| Comp7 | 1.29724 | 0.065908 | 0.0405 | 0.5002 |
| Comp8 | 1.23133 | 0.15731 | 0.0385 | 0.5387 |
| Comp9 | 1.07402 | 0.028533 | 0.0336 | 0.5723 |
| Comp10 | 1.04549 | 0.01923 | 0.0327 | 0.605 |
| Comp11 | 1.02626 | 0.050646 | 0.0321 | 0.637 |
| Comp12 | 0.975611 | 0.046856 | 0.0305 | 0.6675 |
| Comp13 | 0.928755 | 0.063547 | 0.029 | 0.6965 |
| Comp14 | 0.865208 | 0.038095 | 0.027 | 0.7236 |
| Comp15 | 0.827112 | 0.059109 | 0.0258 | 0.7494 |
| Comp16 | 0.768003 | 0.031696 | 0.024 | 0.7734 |
| Comp17 | 0.736307 | 0.027306 | 0.023 | 0.7964 |
| Comp18 | 0.709001 | 0.062685 | 0.0222 | 0.8186 |
| Comp19 | 0.646316 | 0.019582 | 0.0202 | 0.8388 |
| Comp20 | 0.626734 | 0.02443 | 0.0196 | 0.8584 |
| Comp21 | 0.602304 | 0.028955 | 0.0188 | 0.8772 |
| Comp22 | 0.573349 | 0.041167 | 0.0179 | 0.8951 |
| Comp23 | 0.532182 | 0.03834 | 0.0166 | 0.9117 |
| Comp24 | 0.493843 | 0.028593 | 0.0154 | 0.9272 |
| Comp25 | 0.465249 | 0.037692 | 0.0145 | 0.9417 |
| Comp26 | 0.427557 | 0.026056 | 0.0134 | 0.9551 |
| Comp27 | 0.401501 | 0.02385 | 0.0125 | 0.9676 |
| Comp28 | 0.377651 | 0.032733 | 0.0118 | 0.9794 |
| Comp29 | 0.344918 | 0.149131 | 0.0108 | 0.9902 |
| Comp30 | 0.195787 | 0.077867 | 0.0061 | 0.9963 |
| Comp31 | 0.11792 | 0.11792 | 0.0037 | 1 |
| Comp32 | 0 | . | 0 | 1 |

| Supplementary Table 2. Ordinal logistic regression models for the association between caregiver experience components and frailty and sleep disruption, by sex | | | | |
| --- | --- | --- | --- | --- |
| Outcome measures | Men | | Women | |
|  | OLO (95% CI) | p | OLO (95% CI) | p |
| *Frailty measure outcome* | | | | |
| General burden | 1.20 (0.98, 1.47) | 0.080 | 0.99 (0.85, 1.16) | 0.943 |
| Positive emotions | 0.90 (0.70, 1.15) | 0.398 | 0.84 (0.71, 1.00) | 0.056 |
| Financial-led burden | 0.93 (0.62, 1.38) | 0.710 | 1.24 (0.90, 1.71) | 0.186 |
| *Sleep interruption* | | | | |
| General burden | 1.37 (0.68, 2.75) | 0.379 | 2.49 (1.42, 4.36) | 0.001 |
| Positive emotions | 0.99 (0.33, 2.96) | 0.979 | 0.74 (0.60, 0.90) | 0.003 |
| Financial-led burden | 14.42 (1.20, 172.70) | 0.035 | 1.92 (1.21, 3.04) | 0.006 |
| *Sleep trouble* | | | | |
| General burden | 0.93 (0.72, 1.20) | 0.556 | 1.21 (1.01, 1.44) | 0.038 |
| Positive emotions | 0.96 (0.68, 1.34) | 0.788 | 1.09 (0.91, 1.29) | 0.350 |
| Financial-led burden | 1.50 (0.94, 2.38) | 0.089 | 0.91 (0.66, 1.26) | 0.562 |
| *Notes*. Model adjusted for age, education, depression and anxiety symptoms, medical conditions.  Frailty: men, *n* = 94; population, *n* = 759,709; women, *n* = 186; population, *n* = 1,602,631.  Sleep interruption: men, *n* = 25; population, *n* = 189,360; women, *n* = 64; population, *n* = 296,251.  Sleep trouble: men, *n* = 94; population, *n* = 759,709; women, *n* = 186; population, *n* = 1,602,631. | | | | |
